# A Uniform Genomic Minor Histocompatibility Antigen Typing Methodology and Database Designed to Facilitate Clinical Applications

**DOI:** 10.1371/journal.pone.0000042

**Published:** 2006-12-20

**Authors:** Eric Spierings, Jos Drabbels, Matthijs Hendriks, Jos Pool, Marijke Spruyt-Gerritse, Frans Claas, Els Goulmy

**Affiliations:** Department of Immunohematology and Blood Transfusion, Leiden University Medical Center Leiden, Netherlands; Sanofi-Aventis, United States of America

## Abstract

**Background:**

Minor Histocompatibility (H) antigen mismatches significantly influence the outcome of HLA-matched allogeneic stem cell transplantation. The molecular identification of human H antigens is increasing rapidly. In parallel, clinical application of minor H antigen typing has gained interest. So far, relevant and simple tools to analyze the minor H antigens in a quick and reliable way are lacking.

**Methodology and Findings:**

We developed a uniform PCR with sequence-specific primers (PCR-SSP) for 10 different autosomal minor H antigens and H-Y. This genomic minor H antigen typing methodology allows easy incorporation in the routine HLA typing procedures. DNA from previously typed EBV-LCL was used to validate the methodology. To facilitate easy interpretation for clinical purposes, a minor H database named dbMinor (http://www.lumc.nl/dbminor) was developed. Input of the minor H antigen typing results subsequently provides all relevant information for a given patient/donor pair and additional information on the putative graft-versus-host, graft-versus-tumor and host-versus-graft reactivities.

**Significance:**

A simple, uniform and rapid methodology was developed enabling determination of minor H antigen genotypes of all currently identified minor H antigens. A dbMinor database was developed to interpret the genomic typing for its potential clinical relevance. The combination of the minor H antigen genomic typing methodology with the online dbMinor database and applications facilitates the clinical application of minor H antigens anti-tumor targets after stem cell transplantation.

## Introduction

Minor Histocompatibility (H) antigens crucially affect the outcome of HLA-matched allogeneic stem cell transplantation (SCT) [1]. The influence of minor H antigen disparities between HLA identical SCT donor and recipient on graft-versus-host disease (GvHD), graft rejection, and on the graft-versus tumor (GvT) effect has been extensively reported. [2]–[4]. Recently, a new feature of minor H antigens was added comprising its potential to induce and maintain minor H antigen specific T regulator cells after an HLA identical but minor H antigen mismatched renal transplant [5]. The number of molecularly identified minor H antigens has significantly expanded recently, reaching a total of 10 autosomally encoded [6]–[14] and 10 Y-chromosome encoded antigens [15]–[25]. Genomic typing has been developed for some minor H antigens and implemented into the routine procedures of molecular HLA typing. The protocols used however vary and include: allele-specific PCR with sequence specific primers (PCR-SSP) (HA-1 [26], HA-2 [27], and HA-3 [12]), PCR-RFLP (HA-1 [28], HA-8 [9], and HB-1 [8]), gene specific PCR-SSP (UGT2B17 [11] and H-Y [29]), and the use of fluorogenic 3′-minor groove binding probes (ACC-1 [30]). The heterogeneity of these techniques as well as the lack of a molecular typing protocol for some of the molecularly identified minor H antigens prevent their clinical application. Additionally, the interpretation of minor H antigen typing data is relatively complex. Typically, a minor H antigen may differ with respect to tissue distribution and HLA restriction molecule. Some minor H antigen disparities are stimulating the beneficial GvT effect while others are involved in inducing GvHD [31]. Accordingly, pre-transplant information on minor H antigens may be of benefit to the patient. For example, typing of broadly expressed minor H antigen identifies those recipients who are at risk for GvHD. Information on minor H antigens with tissue distribution restricted to the hematopoietic system, e.g. HA-1, HA-2, HB-1, ACC-1, ACC-2, UGT2B17, PANE1, and SP110 [8], [10], [11], [13], [14], [32], is crucial for their potential use in the curative effect GvL of SCT. Likewise, minor H antigens with restricted tissue expression to solid tumors are of relevance to enhance the GvT effect after SCT [33], [34].

Both the expansion of the genomic identification of minor H antigens and the growing interest in the application of minor H antigens as therapeutic tools require a simple and uniform allele specific PCR protocol, an up-to-date database, and tools that facilitate interpretation of the combined HLA and minor H antigen typing data. Here we report on the development of a uniform methodology for genotyping of all currently molecularly identified minor H antigens. Moreover, a minor H antigen database has been developed providing immediate information of the potential clinical relevance of the minor H antigen typing results for the relevant donor/recipient pair.

## Materials and methods

### Development of a uniform genomic minor H antigen typing methodology

In total PCR-SSP for ten autosomally encoded minor H antigens and one for the Y-chromosome encoded H-Y minor H antigen were prepared. The design of the allele specific primers for PCR-SSP (Table 1) was based on the relevant minor H antigen genomic sequences and data from dbSNP [35]. Since typing for minor H antigens will generally be performed in HLA tissue typing laboratories, we developed the minor H antigen PCR protocol based on the most commonly used HLA typing protocols [36]. To this end, primers with a Tm of 62°C were designed. Nucleotide sequences of the primers are listed in Table 1. For HA-1, one 3′ nucleotide was removed from the previously published forward primers [37]. The reported 3′ primers for HA-2 [27] were shortened by 2 nucleotides on the 5′ end. The primers that were earlier described for HA-3 and H-Y matched the proposed protocol [12], [27], [29]. The generated primers for the alleles of HA-1, HA-2, HA-3, HA-8, ACC-2, SP110 and PANE1 specifically amplified the correct allele (Table 2). To establish specificity of the primers for the HB-1 and ACC-1 alleles, mismatches to the gene sequences were introduced in the primer sequences (shaded nucleotides in Table 1).

**Table 1:**
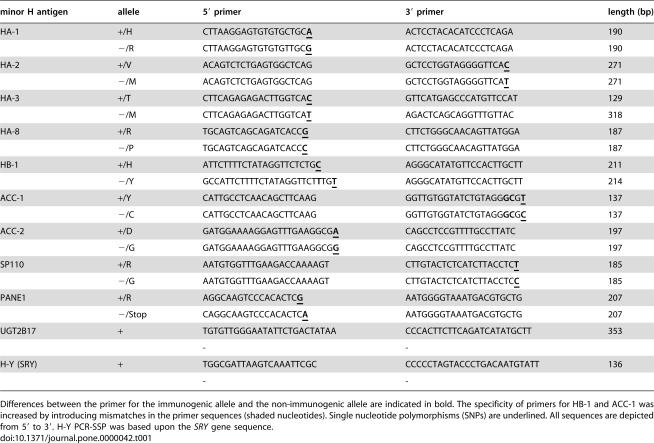
PCR primers for allelic typing of minor H antigens.

minor H antigen	allele	5′ primer	3′ primer	length (bp)
HA-1	+/H	CTTAAGGAGTGTGTGCTGC**A**	ACTCCTACACATCCCTCAGA	190
	−/R	CTTAAGGAGTGTGTGTTGC**G**	ACTCCTACACATCCCTCAGA	190
HA-2	+/V	ACAGTCTCTGAGTGGCTCAG	GCTCCTGGTAGGGGTTCA**C**	271
	−/M	ACAGTCTCTGAGTGGCTCAG	GCTCCTGGTAGGGGTTCA**T**	271
HA-3	+/T	CTTCAGAGAGACTTGGTCA**C**	GTTCATGAGCCCATGTTCCAT	129
	−/M	CTTCAGAGAGACTTGGTCA**T**	AGACTCAGCAGGTTTGTTAC	318
HA-8	+/R	TGCAGTCAGCAGATCACC**G**	CTTCTGGGCAACAGTTATGGA	187
	−/P	TGCAGTCAGCAGATCACC**C**	CTTCTGGGCAACAGTTATGGA	187
HB-1	+/H	ATTCTTTTCTATAGGTTCTCTG**C**	AGGGCATATGTTCCACTTGCTT	211
	−/Y	GCCATTCTTTTCTATAGGTTCT**T**TG**T**	AGGGCATATGTTCCACTTGCTT	214
ACC-1	+/Y	CATTGCCTCAACAGCTTCAAG	GGTTGTGGTATCTGTAGG**GC**G**T**	137
	−/C	CATTGCCTCAACAGCTTCAAG	GGTTGTGGTATCTGTAGG**GC**G**C**	137
ACC-2	+/D	GATGGAAAAGGAGTTTGAAGGCG**A**	CAGCCTCCGTTTTGCCTTATC	197
	−/G	GATGGAAAAGGAGTTTGAAGGCG**G**	CAGCCTCCGTTTTGCCTTATC	197
SP110	+/R	AATGTGGTTTGAAGACCAAAAGT	CTTGTACTCTCATCTTACCTC**T**	185
	−/G	AATGTGGTTTGAAGACCAAAAGT	CTTGTACTCTCATCTTACCTC**C**	185
PANE1	+/R	AGGCAAGTCCCACACTC**G**	AATGGGGTAAATGACGTGCTG	207
	−/Stop	CAGGCAAGTCCCACACTC**A**	AATGGGGTAAATGACGTGCTG	207
UGT2B17	+	TGTGTTGGGAATATTCTGACTATAA	CCCACTTCTTCAGATCATATGCTT	353
		-	-	
H-Y (SRY)	+	TGGCGATTAAGTCAAATTCGC	CCCCCTAGTACCCTGACAATGTATT	136
		-	-	

Differences between the primer for the immunogenic allele and the non-immunogenic allele are indicated in bold. The specificity of primers for HB-1 and ACC-1 was increased by introducing mismatches in the primer sequences (shaded nucleotides). Single nucleotide polymorphisms (SNPs) are underlined. All sequences are depicted from 5′ to 3′. H-Y PCR-SSP was based upon the *SRY* gene sequence.

**Table 2:**
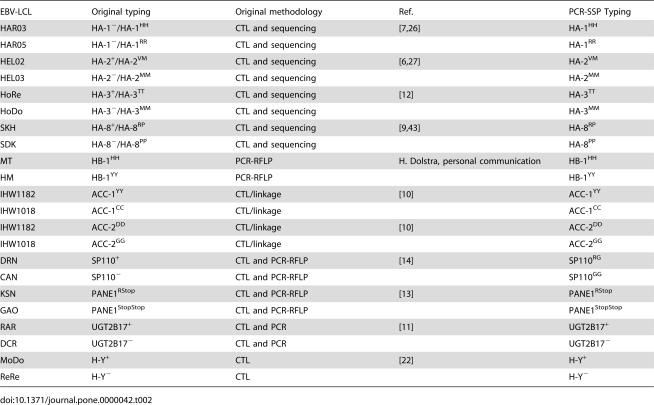
Validation of the PCR-SSP minor H antigen typing methodology.

EBV-LCL	Original typing	Original methodology	Ref.	PCR-SSP Typing
HAR03	HA-1^−^/HA-1^HH^	CTL and sequencing	[7], [26]	HA-1^HH^
HAR05	HA-1^−^/HA-1^RR^	CTL and sequencing		HA-1^RR^
HEL02	HA-2^+^/HA-2^VM^	CTL and sequencing	[6], [27]	HA-2^VM^
HEL03	HA-2^−^/HA-2^MM^	CTL and sequencing		HA-2^MM^
HoRe	HA-3^+^/HA-3^TT^	CTL and sequencing	[12]	HA-3^TT^
HoDo	HA-3^−^/HA-3^MM^	CTL and sequencing		HA-3^MM^
SKH	HA-8^+^/HA-8^RP^	CTL and sequencing	[9], [43]	HA-8^RP^
SDK	HA-8^−^/HA-8^PP^	CTL and sequencing		HA-8^PP^
MT	HB-1^HH^	PCR-RFLP	H. Dolstra, personal communication	HB-1^HH^
HM	HB-1^YY^	PCR-RFLP		HB-1^YY^
IHW1182	ACC-1^YY^	CTL/linkage	[10]	ACC-1^YY^
IHW1018	ACC-1^CC^	CTL/linkage		ACC-1^CC^
IHW1182	ACC-2^DD^	CTL/linkage	[10]	ACC-2^DD^
IHW1018	ACC-2^GG^	CTL/linkage		ACC-2^GG^
DRN	SP110^+^	CTL and PCR-RFLP	[14]	SP110^RG^
CAN	SP110^−^	CTL and PCR-RFLP		SP110^GG^
KSN	PANE1^RStop^	CTL and PCR-RFLP	[13]	PANE1^RStop^
GAO	PANE1^StopStop^	CTL and PCR-RFLP		PANE1^StopStop^
RAR	UGT2B17^+^	CTL and PCR	[11]	UGT2B17^+^
DCR	UGT2B17^−^	CTL and PCR		UGT2B17^−^
MoDo	H-Y^+^	CTL	[22]	H-Y^+^
ReRe	H-Y^−^	CTL		H-Y^−^

### Genomic DNA samples

EBV-LCL typed for HA-8, SP110, PANE1, and UGT2B17 were provided by Dr. E. Warren, Seattle, USA. For ACC-1 and ACC-2, EBV-LCL from individuals from the CEPH (Centre d'Etude du Polymorphisme Humain) panel [10]. The CEPH cell lines have been used earlier by several researchers for identifying novel minor H antigens [10], [38] and are generally available via the Coriell Cell Repositories (http://www.coriell.org; Coriell Institute for Medical Research, Camden, NJ). All cell lines were typed earlier by DNA sequencing, PCR-RFLP, PCR, CTL recognition, or by a combination of CTL recognition and genomic linkage analysis (Table 2). DNA of all samples was isolated using the Chemagic automated magnetic bead technology platform (Chemagen AG, Baesweiler, Germany) according to the recommendations of the manufacturer.

### Genomic PCR

PCR mixes contained 80 ng genomic DNA, 0.25 U Amplitaq (Perkin-Elmer, Norwalk, CT), 0.01% gelatin, 0.8 mM dNTP, 0.5 µM specific primers, 1.5 mM MgCl_2_, 50 mM KCl, 10 mM Tris HCl (pH 8.3), 6% sucrose, and 1 mM cresol red. To increase the yield of HA-1 and HB-1 products, primer concentrations were doubled. The PCR program started with 2 minutes at 94°C, followed by 10 cycles of 10 seconds at 94°C, and 60 seconds at 65°C. Subsequently, 20 cycles were run using the following conditions: 10 seconds at 94°C, 50 seconds at 61°C, and 30 seconds at 72°C. Samples were analyzed on a 2% agarose gel. Internal control primers for human platelet antigen (5′-ACCTAGATAGGTGCGAGCTCACC-3′ and 5′-CAGACTGAGCTTCTCCAGCTTGG-3′; 0.125 µM each) were used for the immunogenic alleles, resulting in a product of 439 bp. For the non-immunogenic alleles, the human growth hormone-2 control primers 5′-CAGTGCCTTCCCAACCATTCCCTTA-3′ and 5′-ATCCACTCACGGATTTCTGTTGTGTTTC-3′ were used to amplify a product of 504 bp. Internal control primers for UGT2B17 had the sequence 5′-TGCCAAGTGGAGCACCCAA-3′ and 5′-GCATCTTGCTCTGTGCAGAT-3′. These primers yield a 761 bp product derived from the HLA-DR locus.

### Minor H antigen database and applications

All currently available information on the molecularly identified minor H antigens has been imported into the minor H antigen database (dbMinor: http://www.lumc.nl/dbminor). Data were retrieved from the relevant literature obtained via PubMed (http://www.ncbi.nlm.nih.gov/entrez), from abstracts in proceedings, or has been forwarded to us. Polymorphisms that have not been functionally characterized as encoding T cell epitopes, as well as minor H antigens that have only been defined at the cellular level, are not included. A ‘submission’ page has been constructed for submitting data on minor H antigen identification directly to the database. Algorithms and queries were developed for searching the dbMinor, for screening the expression of the immunogenic minor H antigenic allele(s) in an HLA-typed individual, and for screening HLA-typed and minor H antigen-typed donor/recipient pairs for putative immune reactivities in the specified combination. The dbMinor database and all applications are available via a web-based interface.

## Results

### Analysis and validation of the uniform genomic minor H antigen typing methodology

**Figure 1:**
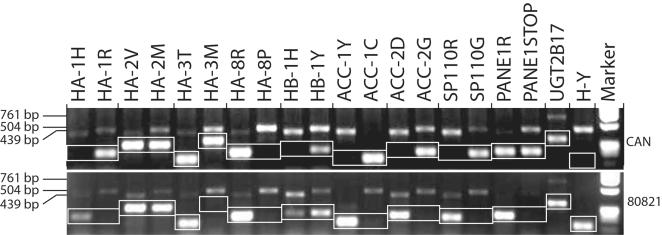
Minor H antigen PCR-SSP. PCR-SSP of DNA of two unrelated individuals (CAN and 80821) was performed and analyzed as described in the Materials and Methods section. The first lane of each minor H locus represents the immunogenic allele; the second lane displays the non-immunogenic allele. Bands relevant for minor H antigen typing are boxed. The products of 439 and 504 bp reflect the internal control for the immunogenic and the non-immunogenic allele respectively. The control band for UGT2B17 has a size of 761 bp. Since the minor H antigens UGT2B17 and H-Y have no allelic counterpart, the latter typing was performed with a single set of primers.

Allele specific PCR-SSP on genomic DNA was established for all minor H antigens and their alleles known to date. Figure 1 displays representative results of the genomic minor H antigen typing by PCR-SSP of 2 EBV-LCL. Clear bands in the correct positions indicate the presence of a minor H antigen allele. Internal control primers demonstrate a band of 439 bp for the immunogenic allele and 504 bp for the non-immunogenic allele. The presence of a minor H antigen allele generally results in a weaker control band. In the absence of the minor H antigen allele, control bands are clearly visible, thus demonstrating correct amplification conditions.

Genomic DNA of 22 reference cell lines was used to validate the newly developed genomic typing method. Table 2 lists the results and shows full concordance with the minor H antigen typing reported previously.

### Minor H antigen genotyping of the CEPH Panel of the 13th IHWS

Upon development and validation of the uniform minor H antigen typing methodology, 22 cell lines from the 13^th^ International Histocompatibility Workshop Symposium (IHWS) reference panel were typed. These cell lines are generally available and have been used before by several researchers to identify novel minor H antigens [10], [38]. The results are depicted in Table 3. These data may serve as standard quality controls for clinical and research laboratories.

**Table 3:**
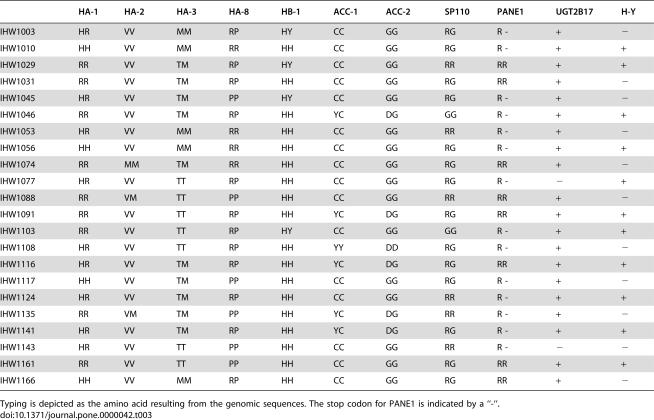
Minor H antigen genotyping of 11 parental CEPH cell lines from the 13^th^ IHIWS.

	HA-1	HA-2	HA-3	HA-8	HB-1	ACC-1	ACC-2	SP110	PANE1	UGT2B17	H-Y
IHW1003	HR	VV	MM	RP	HY	CC	GG	RG	R -	+	−
IHW1010	HH	VV	MM	RR	HH	CC	GG	RG	R -	+	+
IHW1029	RR	VV	TM	RP	HY	CC	GG	RR	RR	+	+
IHW1031	RR	VV	TM	RP	HH	CC	GG	RG	RR	+	−
IHW1045	HR	VV	TM	PP	HY	CC	GG	RG	R -	+	−
IHW1046	RR	VV	TM	RP	HH	YC	DG	GG	R -	+	+
IHW1053	HR	VV	MM	RR	HH	CC	GG	RR	R -	+	−
IHW1056	HH	VV	MM	RR	HH	CC	GG	RG	R -	+	+
IHW1074	RR	MM	TM	RR	HH	CC	GG	RG	RR	+	−
IHW1077	HR	VV	TT	RP	HH	CC	GG	RG	R -	−	+
IHW1088	RR	VM	TT	PP	HH	CC	GG	RR	RR	+	−
IHW1091	RR	VV	TT	RP	HH	YC	DG	RG	RR	+	+
IHW1103	RR	VV	TT	RP	HY	CC	GG	GG	R -	+	+
IHW1108	HR	VV	TT	RP	HH	YY	DD	RG	R -	+	−
IHW1116	HR	VV	TM	RP	HH	YC	DG	RG	RR	+	+
IHW1117	HH	VV	TM	PP	HH	CC	GG	RG	R -	+	−
IHW1124	HR	VV	TM	RP	HH	CC	GG	RR	R -	+	+
IHW1135	RR	VM	TM	PP	HH	YC	DG	RR	R -	+	−
IHW1141	HR	VV	TM	RP	HH	YC	DG	RG	R -	+	+
IHW1143	HR	VV	TT	PP	HH	CC	GG	RR	R -	−	−
IHW1161	RR	VV	TT	PP	HH	CC	GG	RG	RR	+	+
IHW1166	HH	VV	MM	RP	HH	CC	GG	RG	RR	+	−

Typing is depicted as the amino acid resulting from the genomic sequences. The stop codon for PANE1 is indicated by a “-“.

### Minor H antigen database development and applications

**Figure 2:**
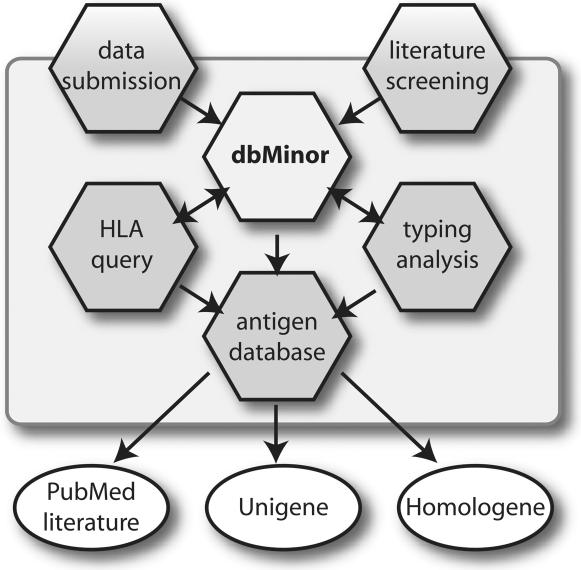
Structure of the minor H antigen database (dbMinor). Data from dbMinor are obtained via direct submission and from literature screening. The data in dbMinor can be analyzed via an HLA-based query, a full typing query and a minor H antigen query. Results from these queries are linked to external data from the NCBI database.

The minor H antigen database (dbMinor) supports 4 applications, i.e. the minor H antigen database (I), the minor H antigen search based upon HLA typing (II), a full donor/recipient minor H antigen and HLA typing analysis (III), and a data submission section (IV). All 4 components of the database and the applications are mutually linked (Figure 2).

#### I. The minor H antigen database

The minor H antigen database allows data searches for basic information on individual minor H antigens. The information includes the references to the relevant articles, the amino-acid sequence of the peptide, and the HLA restriction molecule. If available, links show the related clinical studies, the genes and the protein data available at the NCBI. Likewise, detailed information on tissue distribution and mRNA expression are included.

#### II. The minor H antigen search based upon HLA typing

This searching method has been designed to list the potential expression of minor H antigens in an individual based upon the individuals' HLA typing. HLA typing has to be entered in a two-digit format. The results of the query give an indication whether minor H antigens may play a role in post-transplant immune reactivity and for which minor H antigens typing should be performed. In case HLA sub-alleles have been reported to be unable to express the minor H antigen of interest, it is specifically listed in the results.

#### III. Full donor/recipient minor H antigen and HLA typing analysis

**Figure 3:**
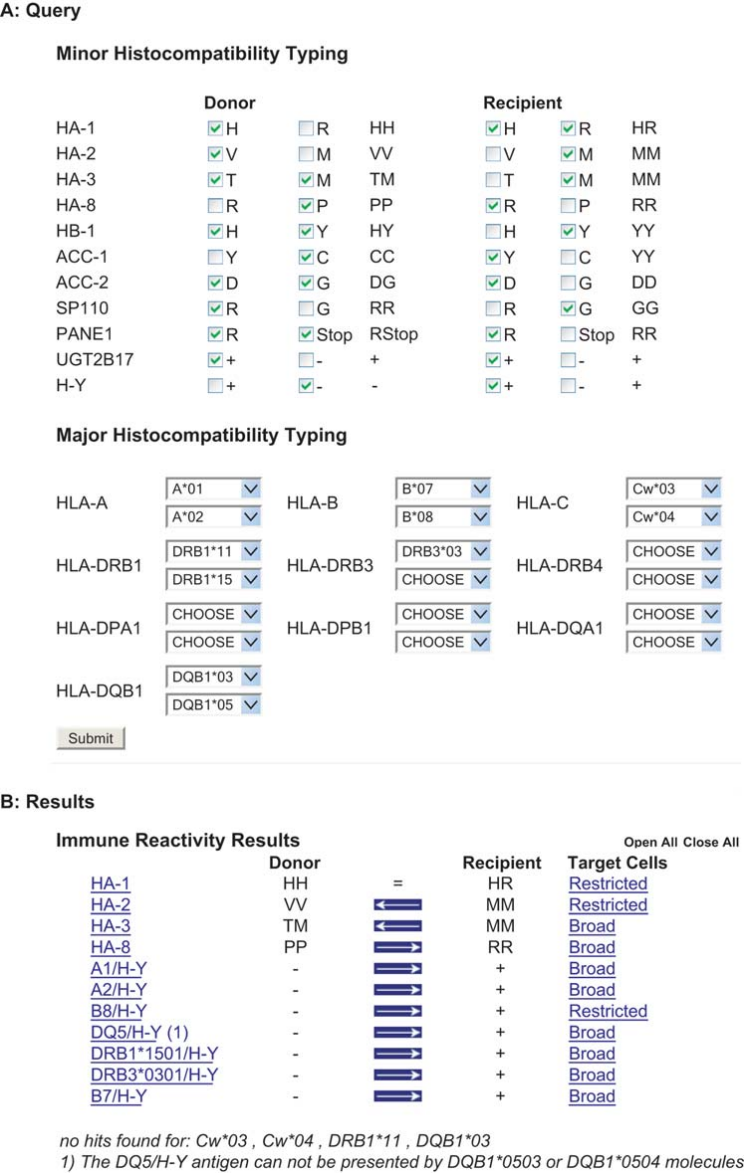
Analysis of donor/recipient HLA and minor H antigen typing. A. Query section: Minor H antigen typing data can be entered by marking the checkboxes per allele per minor H antigen. HLA typing data has the 2-digit format. Full typing for all HLA loci is not required. After submitting the query, a page with results will appear B. Results: The results section lists minor H antigens relevant in the particular donor-recipient combination based upon the HLA typing. In case donor and recipient are minor H antigen matched, as is the case here for HA-1, the sign “ = ” will appear between donor and recipient. In case recipient and donor are minor H antigen mismatched, the disparities are indicated by an arrow. The direction of the arrow indicates the direction of the immune responses. In this example immune reactivity can occur in the recipient-to-donor (host-versus-graft, rejection) direction for HA-2 and HA-3 and in the donor-to-recipient (graft-versus-host) direction for HA-8 and for the various H-Y antigens. The tissue distribution of each relevant minor H antigen is listed as “broad” or “restricted”. By clicking these terms, a detailed list of target cells expressing the minor H antigen will be displayed. Minor H antigens are presented by selected HLA molecules. The HLA alleles expressed by recipient and donor that are not able to present a minor H antigen are listed at the bottom of the results section. Information will appear in case a particular minor H antigen cannot be presented by a specific HLA sub-allele (4-digit format; in this example HLA- DQB1*0503/0504 for DQ5/H-Y).

The combined HLA and minor H antigens typing results of the donor/recipient pair under study subsequently provides information on putative minor H antigen reactivity. An automatic query has been designed to analyze these putative immune reactivities. Upon submission of the locally obtained minor H antigen typing results in combination with the 2-digit HLA typing data (Figure 3a) the algorithm filters all minor H antigens for the HLA restriction molecules expressed in the donor/recipient pair and provides the direction of the potential immune reactivity per individual minor H antigen (Figure 3b). In case the donor and recipient are matched for a relevant minor H antigen, a “ = ” symbol appears. In case of mismatches, the direction of the potential immune reactivity/reactivities is/are indicated with an arrow from recipient to donor in the case rejection and from donor to recipient in the case of GvHD/GvT reactivity. Since a link is created to the tissue distribution of each minor H antigen, clinically relevant information is subsequently provided. If HLA sub-alleles have been reported to be unable to express the minor H antigen of interest, this is specifically listed in the results.

#### IV. Data submission section

New minor H antigen data can be submitted directly to the dbMinor (http://www.lumc.nl/dbminor/submit.html). Submission of information includes local minor H antigen designation, amino acid sequence of the peptide of the immunogenic allele and, if relevant, its allelic counterpart, the HLA restriction molecule, phenotype frequency of the minor H antigen, name of the gene encoding the T cell epitope as assigned by the HUGO Gene Nomenclature Committee, links to Entrez Gene and Homologene, tissue distribution on the cellular and/or RNA level. Optional data that can be submitted are: reference(s) to relevant publications and to clinical studies. If requested, peptide sequence data and protein/gene information can be treated as confidential until publication. The submissions will be generally screened on correctness and made public via the database after final permission of the submitting author. The responsibility of the accuracy of the submitted data remains with the submitting author(s).

## Discussion

Routine typing for minor H antigens of stem cell and solid organ transplant donor and recipient is a prerequisite for their potential clinical use and provides insights in the minor H antigen allo-immune reactivities [39]. A universal and simple methodology enabling genomic minor H antigen typing was, as yet, non-existing. We here describe a uniform protocol for the genomic typing of the currently molecularly identified minor H antigens. Our basic requirements for the development of the method were: 1) easy implementation in routine HLA typing laboratories, 2) high reliability, and 3) optimal reproducibility. To further facilitate implementation in routine HLA tissue typing, PCR primers were designed to run on the most common temperature profile for HLA typing [36]. Choosing separate temperature profiles for each individual minor H antigen might increase the yield of the PCR product. Particularly the PCR-SSP for HA-1 and HB-1 may benefit from such an adaptation. This however also implicates that screening of a single DNA sample would require different PCR runs. Since the yield of HA-1 and HB-1 PCR products is sufficiently high for reliable detection, a single methodology approach is preferred over the optimal amplification. To check its reproducibility, all experiments in this study were performed at least twice, yielding identical results in all experiments (data not shown). Subsequent typing of previously otherwise typed DNA materials demonstrated perfect matching with earlier results (Table 2). Once established, 22 parental CEPH reference cell lines were minor H antigen typed with the developed methodology (Table 3). These typing results can function as reference for the laboratories implementing the minor H antigen typing methodology.

Today, information on minor H antigen typing may have relevant spin-offs in the area of stem cell and renal transplantation. Regarding the former, typing of donor/recipient pairs prior to SCT provides information on two crucial GvH reactivities; i.e.; GvHD and GvT. Broadly expressed minor H antigens are prone to causing GvHD. Pre-SCT information on the disparities for the broad minor H antigens is relevant enabling early anticipation on putative development of GvHD. Minor H antigens with tissue distribution restricted to the hematopoietic system play a role in the graft-versus-leukemia (GvL) effect post-SCT [40]. Typical examples of the latter group are: HA-1, HA-2, HB-1, ACC-1, ACC-2, PANE1 and SP110 [8], [10], [13], [14], [32]. Disparities for these minor H antigens in the GvH direction facilitate the GvL effects of SCT. These GvL effects can be boosted by adoptive immunotherapy using hematopoietic system specific minor H peptides; similarly in the case of SCT for solid tumors the GvT effect can be boosted using minor H peptides expressed on the carcinoma cells but absent on their healthy epithelial counterparts [40].

Beside the development of a uniform genomic typing method for minor H antigens, quick and easy interpretation of the potential clinical consequences of the minor H antigen typing results are desired. Interpretation of the typing results in relation to GvH reactivities in SCT is rather complex; the recipient requires to be homozygous or heterozygous for the immunogenic minor H allele, while the donor should be homozygous for the non-immunogenic allele. Minor H antigens are peptides encoded by polymorphic proteins. In general, only one polymorphic allele of the protein is immunogenic. Some exceptions are reported, e.g. the HLA-B44 restricted HB-1 and the HLA-B60 restricted HA-1 can be immunogenic bidirectionally [41], [42]. Furthermore, minor H antigens are presented in the context of a certain HLA allele. Moreover, as discussed earlier, minor H antigens have different and distinct tissue expression patterns resulting in differential GvH reactivities. Thus, the complex combination of these various characteristics hampers rapid interpretation of the minor H antigen typing results. We therefore developed algorithms to facilitate the analysis of the minor H antigen typing results. This novel application supports in interpreting the typing data with regard to putative minor H antigen specific allo-immune responses and in selecting the correct minor H antigens for adoptive immunotherapy in the relevant donor/recipient pairs.

In all algorithms implemented in dbMinor, the two-digit HLA typing format is used instead of the high resolution 4-digit format. The rationale behind this strategy is lack of complete data regarding this issue for most minor H antigens. For some minor H antigens sub-alleles have been reported that are unable to present the minor H antigen of interest. For example the HLA-DQ5/H-Y minor H antigen can be presented by individuals who carry either the HLA-DQB1*0501 or the HLA-DQB1*0502 allele. However, the HLA-DQB1*0503 and HLA-DQB1*0504 alleles are unable to present this particular H-Y peptide (Laurin et al. in press). If such exceptions have been reported, they are specifically listed. As soon as more information on this issue becomes available for other minor H antigens, 4-digit-based queries will be implemented.

In summary, we here describe a universally applicable genomic PCR-based minor H antigen typing protocol. It offers a simple, uniform, and rapid methodology to determine minor H antigen genotypes of all currently molecularly identified minor H antigens. The methodology can be combined with the online applications for the interpretation of minor H antigen typing results identifying donor/recipient pairs eligible for adoptive immunotherapy and patients with increased risk for developing GvHD. The minor H antigen genomic methodology combined with the online applications will further facilitate the clinical application of minor H antigens in transplantation.

## References

[1] Goulmy E (1996). Human minor histocompatibility antigens.. Curr Opin Immunol.

[2] Goulmy E, Gratama JW, Blokland E, Zwaan FE, van Rood JJ (1983). A minor transplantation antigen detected by MHC-restricted cytotoxic T lymphocytes during graft-versus-host disease.. Nature.

[3] Goulmy E, Termijtelen A, Bradley BA, van Rood JJ (1976). Alloimmunity to human H-Y.. Lancet.

[4] Goulmy E (1997). Human minor histocompatibility antigens: new concepts for marrow transplantation and adoptive immunotherapy.. Immunol Rev.

[5] Cai J, Lee J, Jankowska-Gan E, Derks R, Pool J (2004). Minor H Antigen HA-1-specific Regulator and Effector CD8+ T Cells, and HA-1 Microchimerism, in Allograft Tolerance.. J Exp Med.

[6] den Haan JM, Sherman NE, Blokland E, Huczko E, Koning F (1995). Identification of a graft versus host disease-associated human minor histocompatibility antigen.. Science.

[7] den Haan JM, Meadows LM, Wang W, Pool J, Blokland E (1998). The minor histocompatibility antigen HA-1: a diallelic gene with a single amino acid polymorphism.. Science.

[8] Dolstra H, Fredrix H, Maas F, Coulie PG, Brasseur F (1999). A human minor histocompatibility antigen specific for B cell acute lymphoblastic leukemia.. J Exp Med.

[9] Brickner AG, Warren EH, Caldwell JA, Akatsuka Y, Golovina TN (2001). The immunogenicity of a new human minor histocompatibility antigen results from differential antigen processing.. J Exp Med.

[10] Akatsuka Y, Nishida T, Kondo E, Miyazaki M, Taji H (2003). Identification of a Polymorphic Gene, BCL2A1, Encoding Two Novel Hematopoietic Lineage-specific Minor Histocompatibility Antigens.. J Exp Med.

[11] Murata M, Warren EH, Riddell SR (2003). A Human Minor Histocompatibility Antigen Resulting from Differential Expression due to a Gene Deletion.. J Exp Med.

[12] Spierings E, Brickner AG, Caldwell JA, Zegveld S, Tatsis N (2003). The minor histocompatibility antigen HA-3 arises from differential proteasome-mediated cleavage of the lymphoid blast crisis (Lbc) oncoprotein.. Blood.

[13] Brickner AG, Evans AM, Mito JK, Xuereb SM, Feng X (2006). The PANE1 gene encodes a novel human minor histocompatibility antigen that is selectively expressed in B-lymphoid cells and B-CLL.. Blood.

[14] Warren EH, Vigneron NJ, Gavin MA, Coulie PG, Stroobant V (2006). An antigen produced by splicing of noncontiguous peptides in the reverse order.. Science.

[15] Wang W, Meadows LR, den Haan JM, Sherman NE, Chen Y (1995). Human H-Y: a male-specific histocompatibility antigen derived from the SMCY protein.. Science.

[16] Meadows L, Wang W, den Haan JM, Blokland E, Reinhardus C (1997). The HLA-A*0201-restricted H-Y antigen contains a posttranslationally modified cysteine that significantly affects T cell recognition.. Immunity.

[17] Pierce RA, Field ED, den Haan JM, Caldwell JA, White FM (1999). Cutting edge: the HLA-A*0101-restricted HY minor histocompatibility antigen originates from DFFRY and contains a cysteinylated cysteine residue as identified by a novel mass spectrometric technique.. J Immunol.

[18] Vogt MH, de Paus RA, Voogt PJ, Willemze R, Falkenburg JH (2000). DFFRY codes for a new human male-specific minor transplantation antigen involved in bone marrow graft rejection.. Blood.

[19] Warren EH, Gavin MA, Simpson E, Chandler P, Page DC (2000). The human UTY gene encodes a novel HLA-B8-restricted H-Y antigen.. J Immunol.

[20] Vogt MH, Goulmy E, Kloosterboer FM, Blokland E, de Paus RA (2000). UTY gene codes for an HLA-B60-restricted human male-specific minor histocompatibility antigen involved in stem cell graft rejection: characterization of the critical polymorphic amino acid residues for T- cell recognition.. Blood.

[21] Vogt MH, van den Muijsenberg J, Goulmy E, Spierings E, Kluck P (2002). The DBY gene codes for an HLA-DQ5 restricted human male specific minor histocompatibility antigen involved in GvHD.. Blood.

[22] Spierings E, Vermeulen C, Vogt MH, Doerner LEE, Falkenburg JHF (2003). Identification of HLA class II-restricted H-Y-specific T-helper epitope evoking CD4+ T-helper cells in H-Y-mismatched transplantation.. Lancet.

[23] Zorn E, Miklos DB, Floyd BH, Mattes-Ritz A, Guo LX (2004). Minor histocompatibility antigen DBY elicits a coordinated B and T cell response after allogeneic stem cell transplantation.. J Exp Med.

[24] Torikai H, Akatsuka Y, Miyazaki M, Warren EH, Oba T (2004). A novel HLA-A*3303-restricted minor histocompatibility antigen encoded by an unconventional open reading frame of human TMSB4Y gene.. J Immunol.

[25] Ivanov R, Aarts T, Hol S, Doornenbal A, Hagenbeek A (2005). Identification of a 40S ribosomal protein S4 - Derived H-Y epitope able to elicit a lymphoblast-specific cytotoxic T lymphocyte response.. Clinical Cancer Research.

[26] Wilke M, Dolstra H, Maas F, Pool J, Brouwer R (2003). Quantification of the HA-1 gene product at the RNA level; relevance for immunotherapy of hematological malignancies.. Hematol J.

[27] Wilke M, Pool J, Goulmy E (2002). Allele specific PCR for the minor Histocompatibility antigen HA-2.. Tissue Antigens.

[28] Tseng LH, Lin MT, Martin PJ, Pei J, Smith AG (1998). Definition of the gene encoding the minor histocompatibility antigen HA-1 and typing for HA-1 from genomic DNA.. Tissue Antigens.

[29] Lo YMD, Tein MSC, Lau TK, Haines CJ, Leung TN (1998). Quantitative analysis of fetal DNA in maternal plasma and serum: Implications for noninvasive prenatal diagnosis.. American Journal of Human Genetics.

[30] Nishida T, Akatsuka Y, Morishima Y, Hamajima N, Tsujimura K (2004). Clinical relevance of a newly identified HLA-A24-restricted minor histocompatibility antigen epitope derived from BCL2A1, ACC-1, in patients receiving HLA genotypically matched unrelated bone marrow transplant.. British Journal of Haematology.

[31] Spierings E, Goulmy E (2005). Expanding the immunotherapeutic potential of minor histocompatibility antigens.. J Clin Invest.

[32] de Bueger MM, Bakker A, van Rood JJ, Van der Woude F, Goulmy E (1992). Tissue distribution of human minor histocompatibility antigens. Ubiquitous versus restricted tissue distribution indicates heterogeneity among human cytotoxic T lymphocyte-defined non-MHC antigens.. J Immunol.

[33] Klein CA, Wilke M, Pool J, Vermeulen C, Blokland E (2002). The Hematopoietic System-specific Minor Histocompatibility Antigen HA-1 Shows Aberrant Expression in Epithelial Cancer Cells.. J Exp Med.

[34] Fujii N, Hiraki A, Ikeda K, Ohmura Y, Nozaki I (2002). Expression of minor histocompatibility antigen, HA-1, in solid tumor cells.. Transplantation.

[35] Sherry ST, Ward MH, Kholodov M, Baker J, Phan L (2001). dbSNP: the NCBI database of genetic variation.. Nucleic Acids Res.

[36] Olerup O, Zetterquist H (1991). HLA-DRB1*01 Subtyping by Allele-Specific PCR Amplification - A Sensitive, Specific and Rapid Technique.. Tissue Antigens.

[37] Wilke M, Pool J, den Haan JM, Goulmy E (1998). Genomic identification of the minor histocompatibility antigen HA-1 locus by allele-specific PCR.. Tissue Antigens.

[38] de Rijke B, van Horssens-Zoetbrood A, Beekman JM, Otterrud B, Maas F (2005). A frame-shift polymorphism in *P2X5* elicits an allogeneic cytotoxic T lymphocyte response associated with remission of chronic myeloid leukemia.. J Clin Invest.

[39] Goulmy E (2006). Minor histocompatibility antigens: from transplantation problems to therapy of cancer.. Hum Immunol.

[40] Hambach L, Goulmy E (2005). Immunotherapy of cancer through targeting of minor histocompatibility antigens.. Curr Opin Immunol.

[41] Dolstra H, de Rijke B, Fredrix H, Balas A, Maas F (2002). Bi-directional allelic recognition of the human minor histocompatibility antigen HB-1 by cytotoxic T lymphocytes.. Eur J Immunol.

[42] Mommaas B, Kamp J, Drijfhout JW, Beekman N, Ossendorp F (2002). Identification of a Novel HLA-B60-Restricted T Cell Epitope of the Minor Histocompatibility Antigen HA-1 Locus.. J Immunol.

[43] Warren EH, Greenberg PD, Riddell SR (1998). Cytotoxic T-lymphocyte-defined human minor histocompatibility antigens with a restricted tissue distribution.. Blood.

